# Extensive oceanic mesopelagic habitat use of a migratory continental shark species

**DOI:** 10.1038/s41598-022-05989-z

**Published:** 2022-02-07

**Authors:** Matthias Schaber, Sven Gastauer, Boris Cisewski, Nicole Hielscher, Michael Janke, Marian Peña, Serdar Sakinan, James Thorburn

**Affiliations:** 1 Thünen Institute of Sea Fisheries, 27572 Bremerhaven, Germany; 2 Alfred Wegener Institute for Polar and Marine Research, Biologische Anstalt Helgoland, 27498 Helgoland, Germany; 3 Centro Oceanográfico de Baleares (IEO, CSIC), Palma de Mallorca, Spain; 4 Wageningen Marine Research (WMR), 1976 CP Ijmuiden, The Netherlands; 5 Coastal Resource Management Group, Scottish Oceans Institute, University of St. Andrews, St. Andrews, KY16 9AJ UK; 6 Scripps Institution of Oceanography, University of California, San Diego, La Jolla, CA 92037 USA

**Keywords:** Animal migration, Behavioural ecology, Animal behaviour

## Abstract

The identification of movement and behaviour patterns, as well as inter- and intra-population connectivity is crucial in order to implement effective and functional management and conservation measures for threatened migratory species such as tope (*Galeorhinus galeus*). Yet, previous studies struggled to elucidate clear and consistent movement and depth usage patterns of adult tope in the Northeast Atlantic, suggesting a high plasticity in the migration and behaviour. We deployed pop-up satellite archival tags on adult tope during their seasonal summer aggregations in the inner German Bight of the south-eastern North Sea and near a presumed mating site in southwest Scotland. Depth distribution and migration pathways were derived from time series data with location processing. Four individuals followed migration trajectories leaving coastal areas and crossed the European shelf slope into oceanic areas of the Northeast Atlantic, remaining fully pelagic for the rest of the deployment duration. These sharks showed far-ranging migration trajectories and undertook regular and frequent diel vertical migrations, reaching daytime depths of over 700 m. Vertical migration patterns closely overlapped with biological mesopelagic habitat structures and closely tracked the diel migration of organisms from deep scattering layers derived from hydroacoustic recordings. It is hypothesized that adult tope regularly utilize oceanic habitats, foraging on mesopelagic layers in an environment generally considered of low prey density.

## Introduction

The pelagic realms of the earth’s oceans, covering oceanic waters from the surface to 100 m above the seafloor, comprise 99% of the biosphere^1^. Spanning over a wide bathymetric range, the pelagic realm is partitioned into different zones, dependent on the amount of potential light penetration: the Epipelagic zone (0–200 m), the mesopelagic zone (200–1000 m) and the bathypelagic zone (> 1000 m). Contrasting the highly productive coastal zones, the sparse and patchy distribution of prey fields in the epipelagic zone renders it the marine equivalent of a desert. The perceived simple trophic structures of epipelagic open ocean habitats and the scarcity of prey organisms for higher trophic levels are in stark contrast with the biology of the deeper twilight zones of the global oceans. These zones are characterized by a high taxonomic richness, contained within characteristic and ubiquitous acoustic scattering layers that are clearly visible on echosounder displays. Throughout the world oceans with exception of the two poles, these layers consistently occur at depths between 200 and 1000 m. They are believed to sustain both the highest abundance of fish and to constitute the largest total (fish) biomass on earth^2^. Mesopelagic organisms occupy a crucial position in the “biological pump”, i.e. the reprocessing of dissolved and particulate organic matter and play a key role in the vertical coupling between the nutrient poor shallow ocean with the deep ocean habitats and the global biochemical cycling through diel vertical migration (DVM). In terms of biomass, this DVM is considered the largest animal migration on earth^3,4^. The migrations of lower trophic levels connecting these zones have been extensively studied for decades. In recent years, through the advancement of telemetry technology in combination with Argos satellite data, similar behavioural patterns have been observed for higher trophic animals, such as sharks, actively preying on these layers^5^.

Pelagic shark species are disproportionately vulnerable to and threatened by decades of overfishing and bycatch. This functionally important group of sharks is known for wide ranging distributions and migrations and is generally composed of large bodied macropredators feeding high in the food chain^6,7^. The relatively low diversity and scarcity of extant oceanic shark species (in comparison with oceanic bony fishes) suggest that the oceanic realm is not a primary habitat for cartilaginous fishes^8^. A range of investigations on habitat use and behaviour of different oceanic shark species have shown that many of these large epipelagic predators regularly forage on mesopelagic prey. Accordingly, such sharks contribute to the vertical and horizontal nutrient and carbon flux in oceanic habitats. Oceanic whitetip sharks (*Carcharhinus longimanus*), usually distributed in warm surface layers < 200 m, have been shown to undertake excursions into meso- and bathypelagic layers, hypothesized to be related to active foraging^9^. Similar behaviour was shown for other oceanic sharks like shortfin mako (*Isurus oxyrinchus*) or porbeagle (*Lamna nasus*) and often appear to be related to, and constrained by, ambient temperatures^10,11^. Some species were observed to overcome biological constraints of the open seas and physical limitations of deep-water layers by using mesoscale oceanographic features. It was inferred that these movements could be related to feeding on mesopelagic fishes in deep layers^12^.

While many oceanic predators exploit mesopelagic prey resources through deep diving excursions into corresponding depth layers, others have been shown to engage in regular diel vertical migrations, tracking their prey. Such DVM has been shown for a variety of large predatory ecto- and endothermic shark species^13–18^, and distinct diel patterns also occur in large, filter-feeding sharks like basking sharks (*Cetorhinus maximus*) and the megamouth shark (*Megachasma pelagios*)—noting that only the latter seems to fully exploit the total extension of deep scattering layers^19–21^.

Tracking studies highlighted the connectivity of epipelagic and mesopelagic habitats mediated through large predators, and revealed horizontal and vertical movements of sharks in both coastal and offshore environments. Species that display a high plasticity in migration, habitat and behaviour contribute to this connectivity. Basking sharks were shown to seasonally inhabit epipelagic zones of coastal areas while otherwise undertaking large-scale offshore migrations and spending extended periods in the mesopelagic realm^22^. A similar connectivity of coastal and oceanic habitats was also shown for white sharks and tiger sharks^23,24^.

Tope- or school shark- (*Galeorhinus galeus*), listed as Critically Endangered by the IUCN, is considered a “coastal”, medium-sized shark. Tope inhabit cold and warm temperate regions of most major oceans, mainly associated with continental and insular shelves or coastal areas^25^. Five geographically distinct global populations of tope have been identified, with no evidence of connecting gene flow^26^. Within regional populations, oceanographic barriers may restrict substantial gene flow creating regional sub groups^27^.

Generally considered a benthopelagic species with a mainly coastal distribution on continental shelves and slopes, offshore and pelagic distribution in open ocean habitats have also been observed in tope^28–31^. Additionally, tope are considered highly migratory, and variable movement patterns of adult tope within their regional subpopulations have been described, including movements into and across oceanic waters^28,30,32^, partial migration of females between pupping and nursery grounds^31^, residency near known aggregation areas^31^, oceanic migrations between coastal areas^31,33^, large-scale seasonal latitudinal migrations^34,35^ and along-coast migrations^36^. These movements of adult tope are often seasonal in nature and regularly related to their reproductive cycle^31,36^, or shifts in habitat conditions, such as the displacement of warm water masses^34,35^. With periods spent on the continental shelf and occasional excursions in deep-water, some evidence of crepuscular vertical migrations have been presented for tope in deep water areas^29,30^.

Here, first-time observations of adult tope in the Northeast Atlantic, making extensive use of the mesopelagic realm during an oceanic phase of their migration are presented. Engagement in regular DVM behaviour, closely tracking mesopelagic habitat features is demonstrated through a combination of tagging and hydroacoustic data. It is hypothesized that the observed DVM behaviour is related to predation on mesopelagic organisms in regions with otherwise low epipelagic prey availability.

## Results

### The sharks

**Figure 1:**
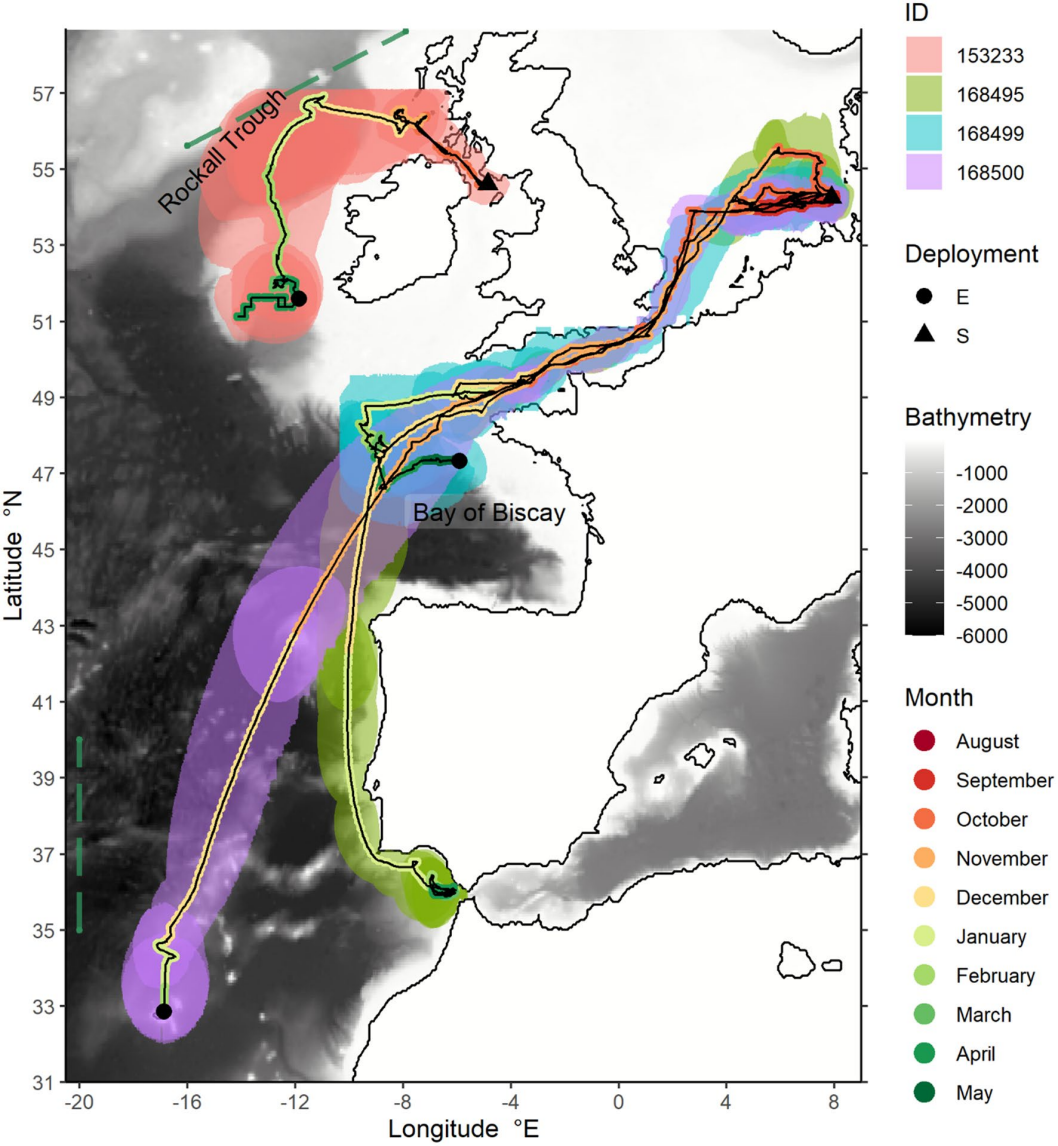
Temporal progression and routes of most likely migrations of four tope (*Galeorhinus galeus*) after deployment of pop-up satellite archival tags in the German Bight of the North Sea (n = 3, 2018 and 2020) and Luce Bay, West Scotland (n = 1, 2015). Most likely trajectories-geolocation from GPE3 state-space model-connect deployment start and end locations (black paths), temporal progression of the tracks colour coded as per the legend. Shaded zones around the most likely tracks colour coded according to tag ID/Ptt indicating 95% location probabilities. Hydroacoustic data used in this study sampled on transects shown as blue-green dashed lines (IBWSS, north, March/April 2016; BATHYPELAGIC, south, June 2018). Bathymetry/Satellite Image data from NOAA/ETOPO1^70^.

As part of a larger migration study, a total of 16 sharks were tagged, out of which four showed clear vertical migration patterns and extensive oceanic habitat use. In the following, focus will be put on these four sharks, whilst a detailed analysis of the horizontal migration patterns is beyond the scope of the present study. One shark was caught and released in southwest Scotland (male, 137 cm TL, shark & tag ID/Ptt 153233) and three sharks were tagged in the German Bight (female, 150 cm TL, ID/Ptt 16845; male, 145 cm TL, ID/Ptt 168499; female, 160 cm TL, ID/Ptt 168500). Tag ID 153233 detached on the 11th April 2016 at 51.59° N 11.86° W in the northern Porcupine Sea bight west of Ireland after the preprogramed 180 days. Tag ID 168500 detached prematurely on the 19th February 2019 at 32.86° N 16.86° W near Madeira Island in the subtropical North-eastern Atlantic after recording 168 days of data. Tag ID 168499 detached a few days short of the preprogramed date on the 14th May 2019 at 47.33° N 5.9° W in the northern Bay of Biscay after recording 253 days of data. Tag ID 168495 also detached short of the preprogramed date on the 24th of April 2021 at 36.12° N 6.07° W on the Atlantic side of the Strait of Gibraltar, recording 223 data days (Fig. 1).

### Migration paths

All sharks remained in the general area where they were caught and released for several weeks, until they started migrating north-westward (southwest Scotland) or westward/south-westward (German Bight). All sharks tagged in the German Bight followed comparatively linear, directed trajectories into the British Channel area, where they remained over a period of a few days to a few weeks, prior to continuing their path further westwards. All four individuals followed trajectories that crossed the European shelf slope into the open, oceanic areas of the Northeast Atlantic. The Scottish shark (ID 153233) resumed an oceanic lifestyle for several weeks until it continued on a southerly trajectory following the slopes of the Porcupine Bank, remaining in that area until detachment of the tag. After crossing into oceanic areas, the sharks tagged in the German Bight followed southerly trajectories. Two individuals (ID 168495 and 168500) moved into the oceanic part of the Bay of Biscay and continued southwards along the Iberian Peninsula, crossing deep water or partly following the shelf slope in a south-westerly direction. Towards the end of the deployment, shark ID 168500 seemed to remain near the insular shelf of Madeira until the tag detached. Similarly, shark ID 168495 remained in the Atlantic part of the Strait of Gibraltar for several weeks until the tag detached. Shark ID 168499 continued on a south-easterly trajectory into the Bay of Biscay and slowly traced the European continental shelf, regularly venturing into oceanic areas. The total estimated trajectory length of the sharks ranged from 2708 km (ID 153233) to 4691 km (ID 168499).

### Depth and habitat use

**Figure 2:**
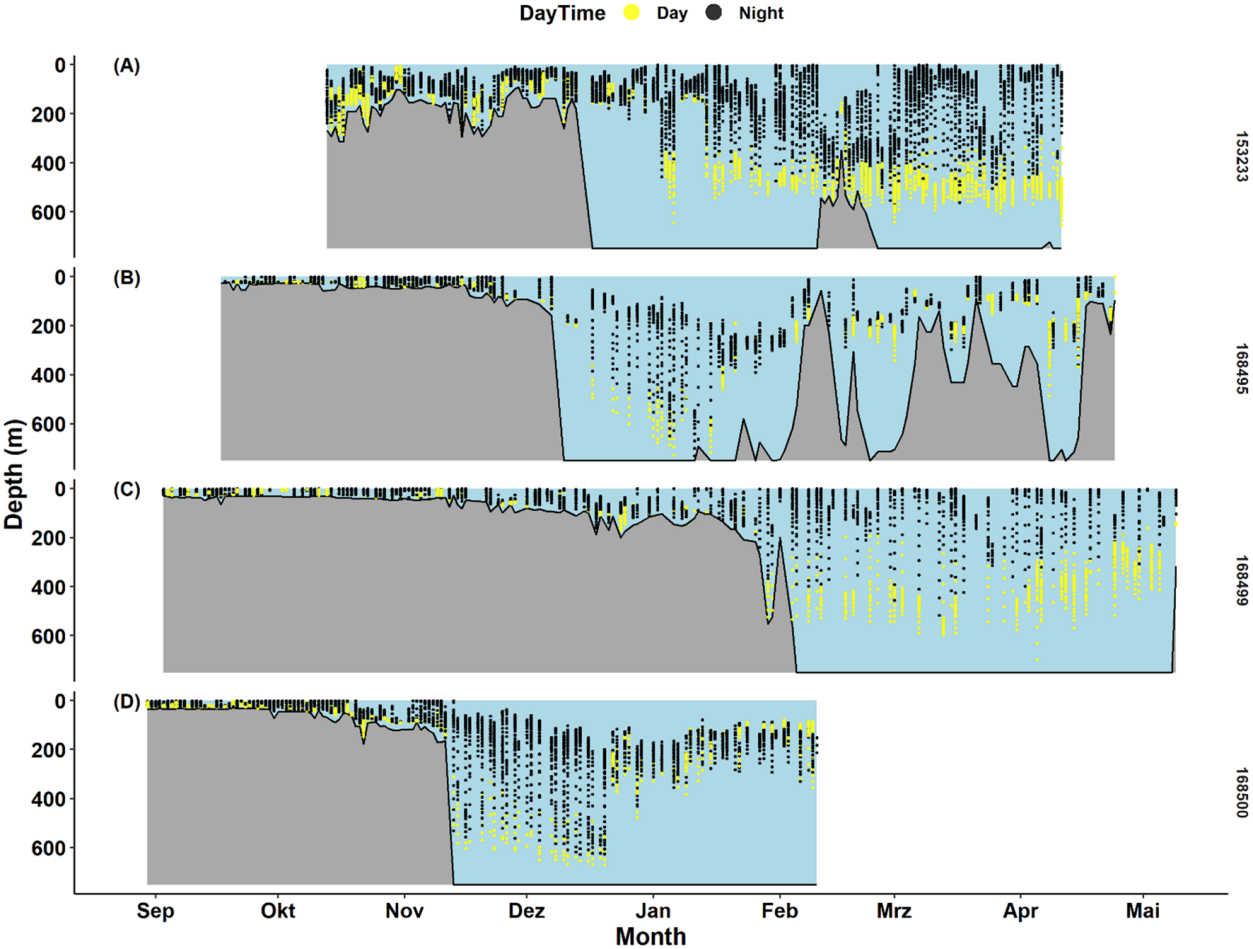
Time series of tope (*Galeorhinus galeus*) depth measurements from PSAT deployments ID 153233 (**A**), ID 168495 (**B**), ID 168499 (**C**) and ID 168500 (**D**). Corresponding bathymetry/seabed depth along the migration path from location processing (GPE3/NOAA ETOPO1) indicated as grey polygon. Due to uncertainties in the geolocation as well as due to the lack of speed and trajectory information for tracked individuals below the surface, the exact bathymetry at the time of a vertical movement is also subject to uncertainty. Yellow dots indicate locations during daytime and black dots, locations during nighttime.

On the continental shelf and in coastal areas, all sharks showed regular vertical movements between near surface layers and close to the seafloor (Fig. 2). No distinct, recurring diel pattern in the vertical distribution was evident (Supplementary Figs. 1–4). The sharks displayed less usage of near surface layers when swimming through on-shelf areas with water depths > 100 m. During the coastal phase, all sharks regularly displayed phases of alternating ascents and descents from the seafloor to surface layers over several tens of meters in comparatively short time intervals.

Once the sharks crossed the continental slope into oceanic waters, they remained pelagic and immediately (exception: ID 153233, Fig. 2A) increased their vertical occupancy range from near surface layers down to 500 m depth and beyond. Maximum recorded depths of the sharks during their oceanic phase ranged from 654 m (ID 153233) to 730 m (ID 168495). In oceanic areas, the sharks displayed regular excursions between surface and deep layers. A regular, periodic pattern over 24 h was evident from these excursions (Supplementary Figs. 1–4). Altogether, the sharks remained in epipelagic layers of mostly at less than 100 m during night-time, moving to deeper layers of around 400–500 m depth during daytime. This pattern of diel vertical migration persisted until the end of the deployments. Occasional fluctuations were observed, where the shark did not ascend to surface layers but remained at depths of 150–200 m during daytime (Fig. 2B,D) or remained in midwater layers of around 200 m depth for day- as well as night-time, e.g. when presumably crossing banks and ridges (Fig. 2A,C). Arriving in proximity of Madeira Island and seemingly following bathymetric features, a slightly different pattern became evident for shark ID 168500. Vertical migrations fluctuated more in terms of maximum depths as well as depth ranges covered and were mostly reduced to the 100–300 m layer (Fig. 2D). Further, after following the continental shelf slope southward, shark ID 168495 abandoned clear DVM behaviour while residing near the Strait of Gibraltar and alternating between coastal and offshore regions (Fig. 2B).

### (Meso)pelagic habitat structure

**Figure 3:**
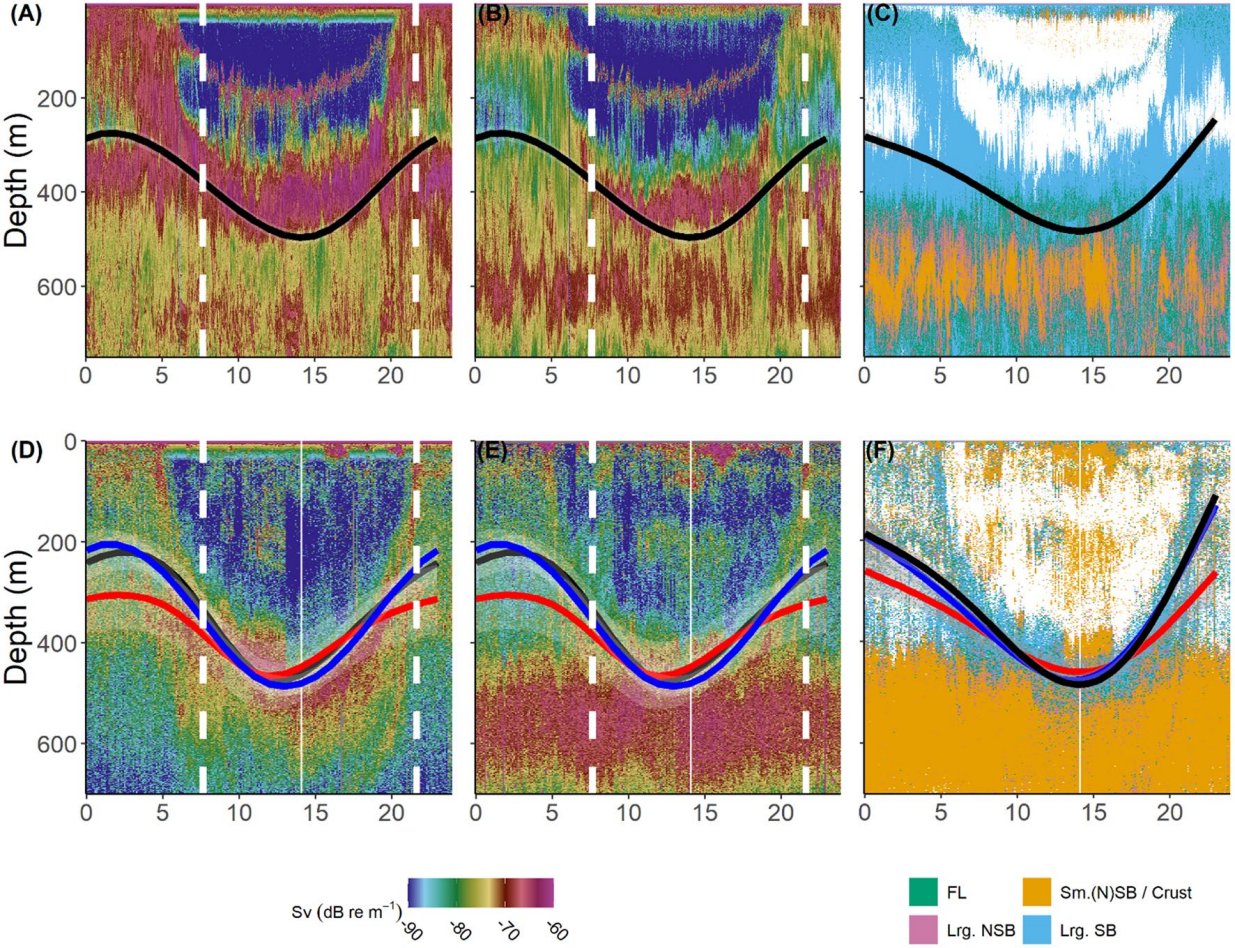
24-h composite echograms of hydroacoustic data (**A**,**D**: Sv 18 kHz; **B**,**E**: Sv 38 kHz; **C**,**F**: Δ_Sv18kHz−38 kHz_ Categories) from IBWSS (temperate Northeast Atlantic, top) and BATHYPELAGIC (subtropical Northeast Atlantic, lower). Overlays: Predicted (GAMM, coloured paths) depths of tope (*Galeorhinus galeus*) shown for shark ID 153233 (IBWSS region, black path, top panels) and sharks ID 168495, ID 168499 and ID 168500 (black, red and blue paths, respectively, lower panels). Curves represent most likely depth position at a given time of the day. Sunrise and sunset indicated as vertical dashed lines (mean time classified from tag time series data during the oceanic phase). White vertical line in the BATHYPELAGIC plots represents missing data.

In both the northern temperate (surveyed by IBWSS) and more southern, subtropical (surveyed by BATHYPELAGIC) oceanic areas of the North East Atlantic, clear mesopelagic structures are prominent features, evident as distinct Sound Scattering Layers (SSL) on echograms. Continuous, persisting and distinct Deep Scattering Layers (DSL) were evident in the acoustic dataset. Both during the IBWSS and the BATHYPELAGIC survey these distinct layers were observed at 18 and 38 kHz. A stationary layer was observed at 400–500 m in the Rockall Trough (north) (Fig. 3A–C) and at 400–600 m in the area of the Azores Front (south) (Fig. 3D–F). The presence of a migrating deep scattering layer (MDSL) was evident in both datasets. The MDSL merged with the upper part of the stationary DSL during daytime, ascending towards surface layers (< 200 m) during night-time. A secondary MDSL with daytime depths of around 200 m and night-time depths of approximately 50 m was observed in the Rockall Trough region.

Differences in acoustic backscatter at the two frequencies allowed structuring of the acoustic water column data into several functional groups. Both MDSLs generally showed higher backscattering values at 18 kHz than at 38 kHz. In contrast, backscattering values were higher at 38 kHz in the non-migrating, stationary deep scattering layers (NMDSL). In the IBWSS (Fig. 3C), the upper part of the stationary NMDSL between 400 and 500 m was dominated by strong echoes classified as large swimbladdered fishes. The joining MDSL showed a similar pattern with some contributions of fluid-like scatterers. At around 500 m, a thin layer where the acoustic backscatter was dominated by organisms with fluid-like backscattering properties (cephalopods and gelatinous zooplankton) followed. The central part of the NMDSL around 600 m depth showed high contributions of acoustic backscatter categorized as small, swimbladdered fishes including small, non-swimbladdered fishes and crustaceans as well as some large, non-swimbladdered fishes. All MDSLs within the IBWSS region were distinctly dominated by large swimbladdered fishes with some contributions of fluid-like scatterers (including cephalopods). The latter, together with large non-swimbladdered fishes, contributed dominantly to the total backscatter in epipelagic layers during daytime. In the BATHYPELAGIC (Fig. 3F), the stationary NMDSL (> 400 m depth) consisted largely of backscatter assumed to be originating mainly from small, non-swimbladdered fishes and crustaceans with negligible contributions of the other categories.

### Diurnal vertical migration: the sharks

**Figure 4:**
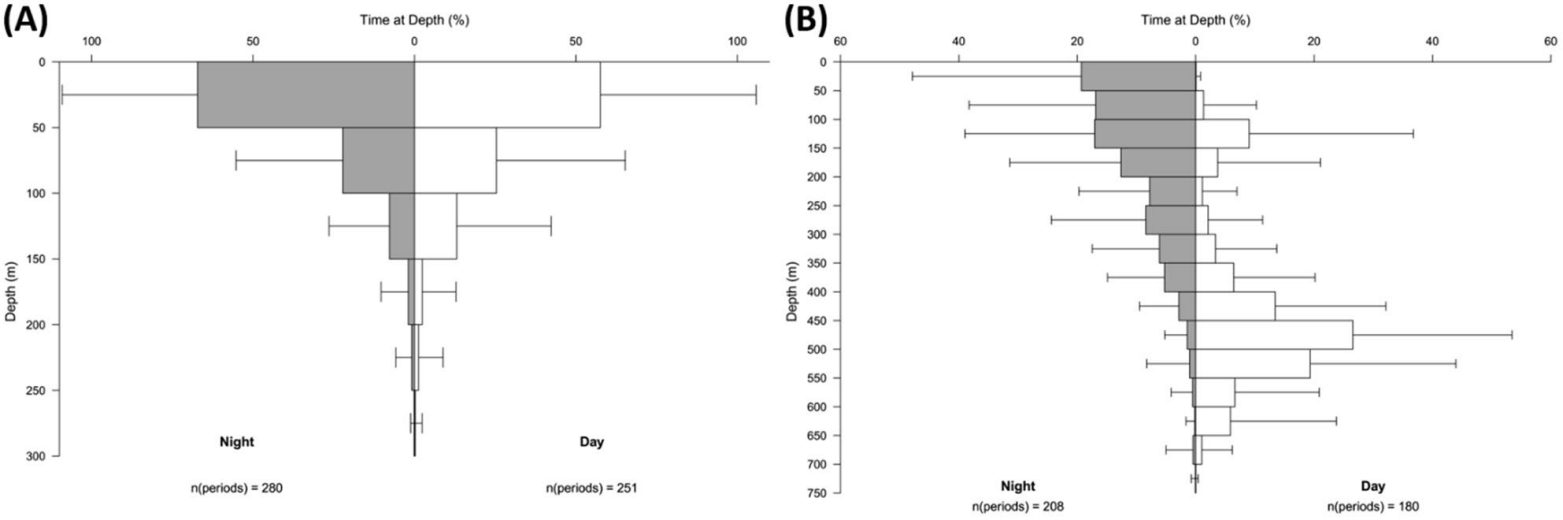
Combined time at depth (TAD) histograms for four tope (*Galeorhinus galeus*) during the coastal/continental (**A**) and oceanic (**B**) parts of their migration.

All four sharks showed clear evidence of temporally and spatially explicit depth and habitat occupancy as well as DVM behaviour. In continental and coastal areas, no apparent diel patterns in depth distribution and habitat use were evident. All sharks spent comparable amounts of time in shallow and deeper regions, during both day and night (Fig. 4).

**Figure 5:**
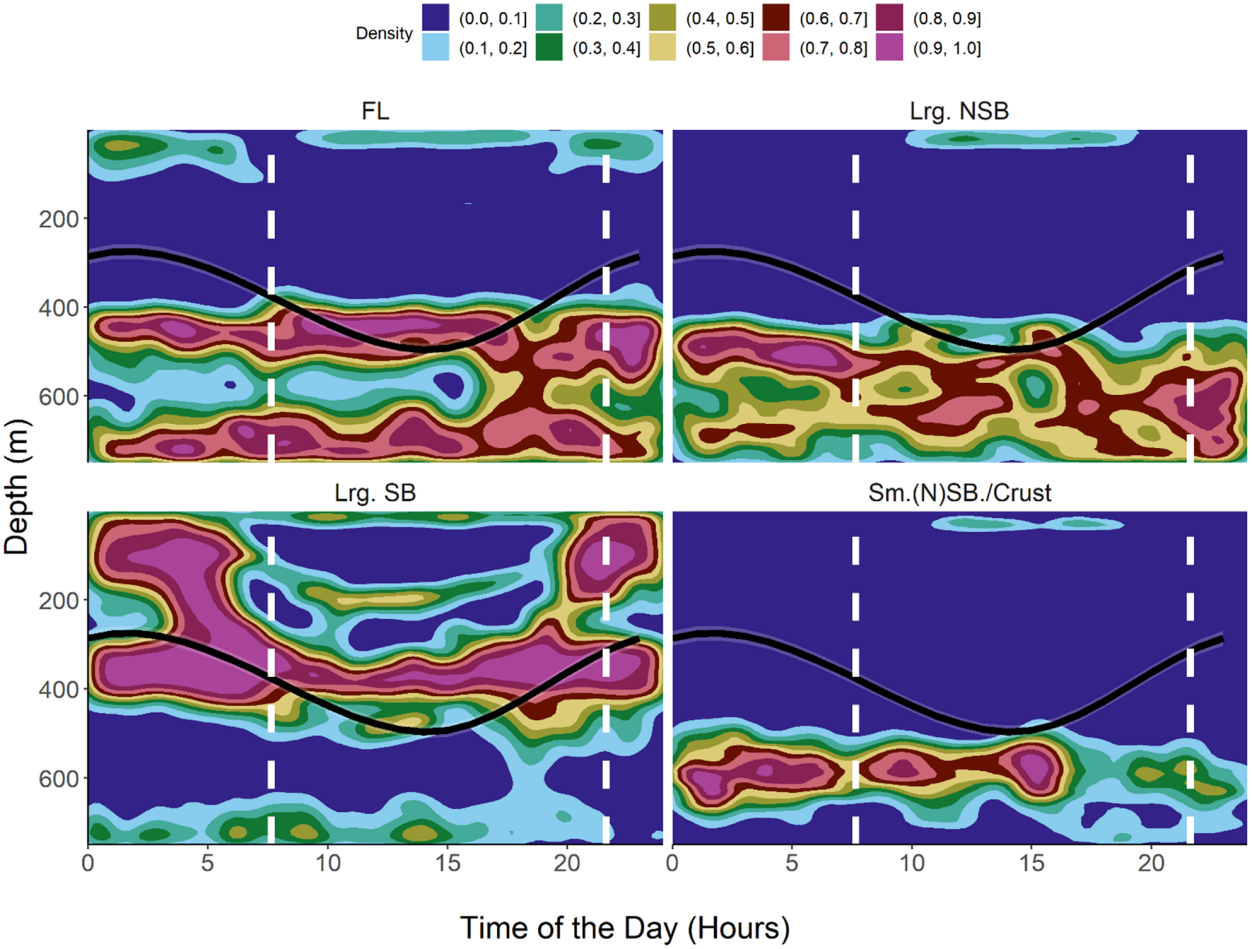
2D kernel density distributions scaled to 1 of different hydroacoustically classified functional groups within the IBWSS survey region of the temperate Northeast Atlantic. Classification based on Δ_Sv18kHz−38 kHz_, scaled to 1, with FL = fluid-like scatterers (cephalopods, gelatinous zooplankton); Lrg. NSB = large non-swimbladdered fishes, Lrg.SB = large swimbladdered fishes and Sm.(N)SB/Crust. = small swimbladdered and non-swimbladdered fishes and crustaceans. Overlays: Predicted (GAMM, black path) depths of tope (*Galeorhinus galeus*) shown for shark ID 153233. Curve represents most likely depth position at a given time of the day. Sunrise and sunset indicated as vertical dashed lines.

**Figure 6:**
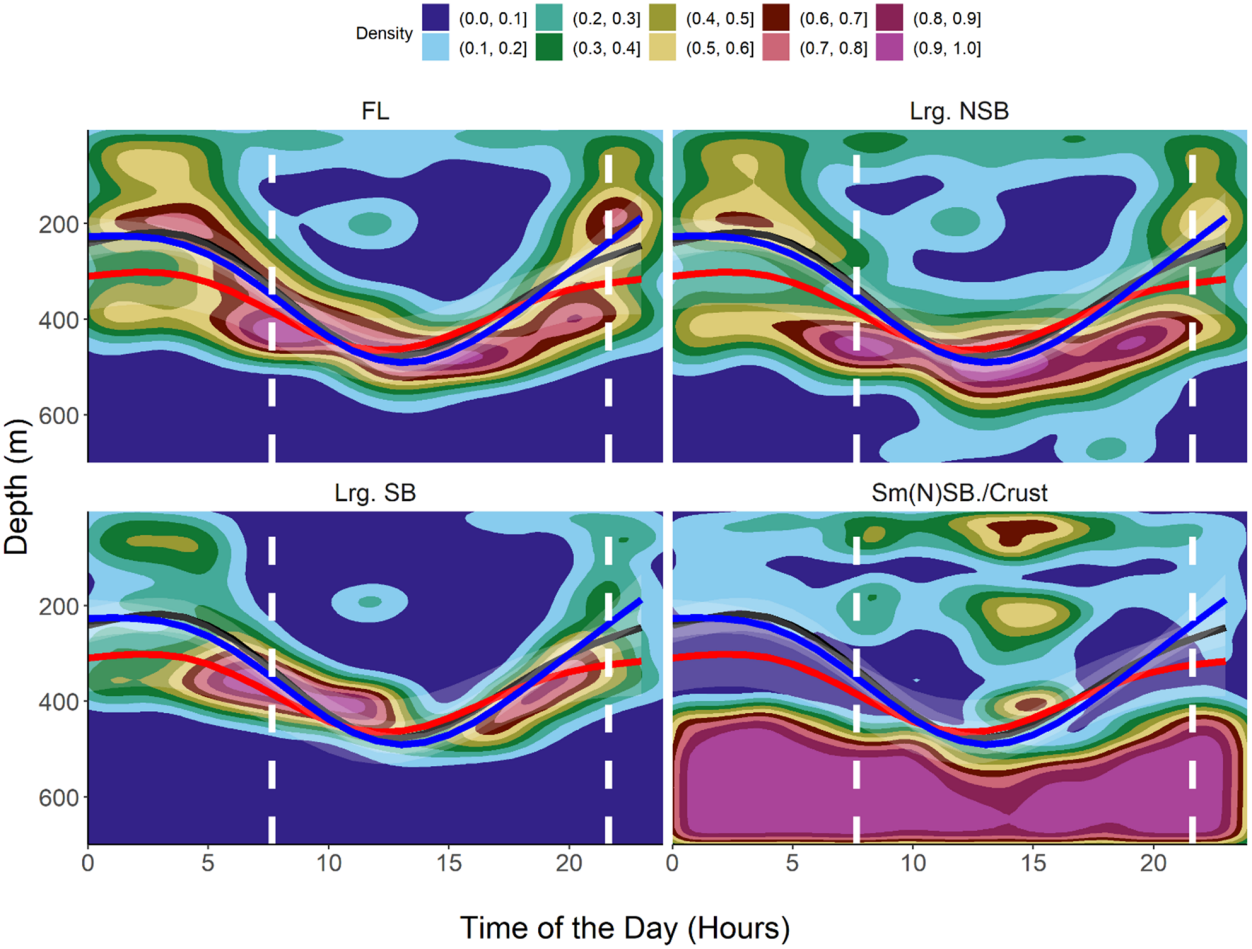
2D kernel density distributions scaled to 1 of different hydroacoustically classified functional groups within the BATHYPELAGIC survey region of the subtropical Northeast Atlantic. Classification based on ΔSv18 kHz–38 kHz, scaled to 1, with FL = fluid-like scatterers (cephalopods, gelatinous zooplankton); Lrg.NSB = large non-swimbladdered fishes, Lrg.SB = large swimbladdered fishes and Sm.(N)SB/Crust. = small swimbladdered and non-swimbladdered fishes and crustaceans. Overlays: Predicted (GAMM, black, red and blue paths) depths of tope (*Galeorhinus galeus*) shown for sharks ID 168495, ID 168499 and ID 168500, respectively. Curves represent most likely depth position at a given time of the day. Sunrise and sunset indicated as vertical dashed lines.

Mean daytime depth in oceanic waters was observed to be 412 m (s.d. = 150 m, N = 13,344) while the mean night-time depth was 174 m (s.d. = 121, N = 20,028). Clear DVM patterns in habitat use and depth distribution were observed during the extended periods of the oceanic phase of the migrations. In open ocean areas, the sharks exhibited a clear and periodic DVM, moving from near-surface during night-time into mesopelagic layers around 400–600 m depth during daytime. The most frequently occupied depth layers during night-time were 0–150 m (the sharks cumulatively spent around 60% of their time in these layers). During daytime for approximately 60% of the time the sharks were observed in depths of 400–600 m. During daytime, little time (10%) was spent around 100–150 m of depth (Fig. 4). Generally, the observed vertical distribution pattern was related to daytime, with the time of the day (in hours) explaining roughly 50% of the variability in depth distribution (Table 1, Figs. 3, 5, 6).

**Table 1:** Model scores: GAMMs are ${Z}_{shark }\sim s\left(Time, k = 5, bs = 'cc'\right)+Ptt$, where Z_shark_ is the depth location of the shark at any given time expressed in rounded hours (Time), k defines the degrees of freedom and s is the Gaussian smoother function. *Ptt* Tag Identifier/Shark, *df* degrees of freedom, *SE* Standard Error, *p* p value.

Ptt/ID	R^2^	df	SE	P
153233	0.46	2.96	6.25–6.74	< 0.01
168500	0.51	2.93	8.68–15.61	< 0.01
168499	0.50	2.92	8.97–12.42	< 0.01
168495	0.47	3.97	2.81–49.23	< 0.01
All	0.45	2.96	0.00–46.61	< 0.01

During daytime, the sharks remained in regions of the MDSL, below 400 m of depth, with strongest backscattering strengths at 18 kHz. Through a DVM behaviour initiated around sunset (ascent) and sunrise (descent), the sharks largely followed the 18 kHz MDSL concurrently emerging from the DSL. This MDSL vertically migrated into shallower layers around 200 m of depth (north) and 100–200 m of depth (south), to later descend and merge with the continuous and permanent NMDSL around sunrise again (Fig. 3). In the IBWSS region (Fig. 5), shark ID 153233 largely remained within the MDSL dominated by the acoustically identified class large swimbladdered fishes during night-time. During daytime the shark descended into the upper part of the NMDSL, where it mostly remained within the layers of highest densities of fluid-like scatterers.

In the southern region (BATHYPELAGIC), the sharks clearly traced the MDSL with highest probabilities of encountering the highest densities of large swimbladdered fishes and cephalopods/gelatinous zooplankton (fluid-like scatterers) (Fig. 6). During their deep daytime residency within upper parts of the NMDSL there was a distinct overlap of the sharks with layers that showed highest kernel densities for both categories. The daily vertical trajectories of the considered sharks largely tracked the vertical path of highest probabilities of encountering the highest available densities of cephalopods and gelatinous zooplankton. In contrast, layers with increased densities of small non-swimbladdered fishes and crustaceans seemed to be avoided.

## Discussion

The importance of the mesopelagic realm for tope is highlighted. A shift in habitat use and foraging behaviour of the sharks is suggested, with the sharks switching from no distinct (diel) patterns in depth use in shelf waters to a strong, likely prey driven DVM behaviour in oceanic areas. A close association with mesopelagic scattering layer structures is evidenced here for the first time. Diel patterns in vertical movements of tope have previously been identified using archival tags showing tope ascending at night and descending into deeper waters during daytime. These observations were hypothesized to be related to feeding, but due to the uncertainty of geolocation estimates from the archival tags used, it could not be determined whether the sharks remained close to the seafloor on deep continental slope areas during daytime or in fact entered pelagic layers of oceanic waters^29^. Additional observations of some tope engaging in diurnal vertical migration while in deep-water habitats of the Northeast Atlantic were also inferred as related to feeding and shifts in feeding strategies, but could not be related to corresponding habitat features or prey fields^30^.

Whilst on the continental shelf, no clear regular diurnal dive patterns were observed, and the sharks mostly used the whole available depth range and water column. In previous studies, weak cyclic patterns of standard and crepuscular diel migrations had been identified when tagged tope remained in comparatively deep areas of circa 250 m^30^. Other studies showed no regular patterns in depth use of adult tope whilst in shallow habitats. In a shallow natural reserve in northern Patagonia, adult tope tagged with PSATs spent an equal amount of time in near-surface layers both day and night, but exhibited “yo yo-dives” towards the seafloor with maximum dive depths recorded at night^37^. The sampling rate of the PSATs used in the present study was insufficient to allow for a clear quantification of diving speeds and behaviours whilst in more coastal waters. The observed alternating ascents and descents from near the seafloor to surface layers and back over a comparatively short time span could possibly be ascribed “yo yo-dives”, but further investigation would be required to clearly identify those as such. Such diving behaviour has also been observed in other shark species and inferred to be an energy efficient swimming strategy^38^ and/or be related to behavioural thermoregulation and prey searching^39,40^. The purpose of the observed dives in the shallower coastal areas is not very clear, occurring within largely well-mixed layers. It is likely that these short dives served as a search strategy to locate prey throughout the water column^40^.

In oceanic areas, regular and recurring diel dive patterns were observed for all four sharks. Such behaviour could be attributed to several drivers such as thermoregulation, navigation, and foraging^9,12,41–44^ or a combination of these. Diving to aid navigational cues cannot be ruled out as some sharks showed distinct and clear, more or less straight-lined migration trajectories in oceanic areas. The close association of the sharks with biological, mesopelagic habitat structures as well as the regularity of the diel dive patterns rule out instantaneous predator avoidance or short-term prey searching to be the driving force behind the observed behaviour. Alternatively, we interpret the clear and regular DVM observed in tope during their oceanic phase and their association with certain functional groups comprising mostly the migrant deep scattering layer (MDSL) but also parts of the non-migrant deep scattering layer (NMDSL) as most likely related to feeding behaviour, exploiting prey from the available mesopelagic communities.

The occurrence of prominent and continuous hydroacoustically measurable DSL consisting of taxonomically highly variable and highly abundant mesopelagic species is an ubiquitous phenomenon observed in deep and oceanic areas of the world’s oceans^2,45,46^. Another unique feature of these layers across all oceans are regular vertical migrations exhibited by many members of this mesopelagic community and covering a large vertical range^4,47,48^. Seasonal information on dynamics of the DSL are scarce, but show that the non-migrant deep scattering layers (NMDSL) generally maintain both vertical distribution and also intensity throughout the year, except in upwelling regions^49–51^. The shallower, migrant layer (MDSL) observed in the Bay of Biscay, through which three of the sharks migrated, seasonally varies in intensity and species composition, but its diel depth structuring can be considered stable (Peña, unpublished data). Both the NMDSL and the MDSL show similar vertical distributions in the Bay of Biscay and the southern area covered by the BATHYPELAGIC survey, and are consistent with the DVM identified for the “southern” sharks. Analogous patterns were observed for the northern IBWSS survey and the “northern” shark. Hence, the corresponding observations from the spatio-temporally seemingly distant hydroacoustic measurements are considered representative and appropriate for describing the mesopelagic habitat structure encountered by the sharks during their oceanic migrations.

The acoustically classified mesopelagic layers mostly hint towards a dominance of teleost fishes, crustaceans, cephalopods and gelatinous zooplankton. Fishes without a swim-bladder, crustaceans and squid yielded a much weaker contribution to the overall recorded backscatter when compared to fishes with a swim-bladder. It is important to note that based on the physical properties of the acoustic waves at the given frequencies and the acoustic impedance of the organisms, gas-bearing organisms are expected to produce a much stronger acoustic backscatter than non-gas bearing organisms^52^. Therefore, if one was to consider numerical abundance values, these “weaker” scatterers likely had a more significant contribution to the DSL^47^. Classification of acoustic targets into functional groups remains challenging, but classification into broad categories based on physical properties and known characteristic acoustic scattering patterns at discrete frequencies allows for a classification with some degree of certainty^53^.

In studies conducted in both, the subtropical eastern Atlantic and the northern Atlantic, mainly myctophid fishes contributed to the MDSL^47,54^. In the stationary NMDSL that showed stronger backscatter at 38 kHz, *Cyclothone* spp. (bristlemouths) were most likely the dominant taxonomic group^47,49^. The species inhabiting these depths (*Cyclothone braueri*) has resonance at 38 kHz, masking the acoustic scattering of migrating organisms at this frequency^48,49^. Cephalopods also comprise an important fraction of both the NMDSL as well as the MDSL^53,55^. Many cephalopod species are known to engage in DVM behaviour^55,56^. Biological validation from alternative sources, complementing the hydroacoustic classification is not available here. Nonetheless, we consider the assumption that, based on the presented acoustic classification, the MDSL is dominated by swimbladdered myctophids with comparatively sparse but consistent contributions of cephalopods, as robust.

The diet of tope mainly consists of teleost fishes and cephalopods, albeit with regionally and temporally varying contributions^32^. In shallow coastal areas of the southwestern Atlantic, adult tope are known to periodically feed mainly on benthic teleosts or on squid whilst avoiding pelagic teleosts. This hints towards tope, occasionally being a selective rather than opportunistic predator^57^. Tope caught on the insular shelf of the Azores archipelago in less than 150 m depth were observed to almost exclusively prey upon schooling pelagic fish (boarfish *Capros aper* and snipefish *Macrorhamphosus scolopax*) with only minimal contributions of cephalopods to the diet^58^. Even though it was shown that pelagic fishes contributed to the diet of tope in coastal areas^59^, especially near oceanic islands, mesopelagic teleosts like lanternfishes (myctophids) have not been identified as a contributing factor in the diet of tope. This could be related to a lack of samples of stomach contents from oceanic tope or possibly faster digestion of these fishes. However, cephalopods have been shown to constitute an important part of tope diet^29,32^. Other high trophic sharks are assumed to specifically exploit cephalopods in oceanic areas, including during deep dives into mesopelagic layers^9,60^. Increased daytime vertical habitat use of silvertip sharks (*Carcharhinus albimarginatus*) has, in combination with their known prey preferences, specifically been associated with DVM of cephalopods^61^. Accordingly, we interpret the observations of tope seemingly following the DVM pattern of lanternfishes and cephalopods as an attempt to maximize squid encountering chances.

The high importance of vertically migrating organisms and especially fishes in the oceanic biological carbon flux (the “biological pump”), cycling carbon between the surface ocean and the mesopelagic zone, connecting nutrient poor shallow ocean with deep ocean habitats, remains poorly understood^62^. While oceanic cephalopods constitute ubiquitous features of migrating mesopelagic communities and also constitute important components of the pelagic food web linking various components of marine ecosystems, their contribution to this energy flow is also poorly understood^63^. Recent analyses on the trophic geography of sharks have shown that oceanic sharks use carbon sourced from productive oceanic areas of a comparatively narrow latitudinal range. Shelf-dwelling sharks on the other hand mostly source their carbon from regional pelagic ecosystems, with a generally broader range in food web utilization. Shelf-dwelling sharks that used multiple habitats and also undertook migrations exceeding 1000 km were still considered to largely assimilate carbon supporting tissue growth from foraging within shelf areas^64^. In contrast, oceanic sharks, often migrating over long distances during their life history, are generally considered oceanic nomads. Nonetheless many of those species occur close to shore, rendering a precise classification as “oceanic nomad” vs. “coastal resident” difficult^10^. Inversely, this also holds validity for species considered coastal or “continental”—like tope.

While an unexpected and regular deep diving behaviour was observed in scalloped hammerhead sharks (*Sphyrna lewini*)—a species considered coastal and semi-oceanic-speculated to be driven by feeding behaviour, preying on mesopelagic fish^65^, no such spatially explicit observations were made in benthopelagic, allegedly coastal shark species like tope. Tope have previously been shown to undertake (regular and partially diurnal) dives into depths of more than 800 m in oceanic areas^29,30^. The exploitation of oceanic prey by tope and their oceanic habitat use, especially during migration as shown in this study, could represent another hitherto disregarded vertical and horizontal link of oceanic and coastal/shelf ecosystems, through energy export during presumably recurring transient movements between on-shelf and oceanic, mesopelagic habitats. Additionally, even though this behaviour was only observed in a small sample of tagged tope, the common traits observed across areas and regions might lead to reconsidering tope from being a mainly coastal and continental species. We have found a relatively consistent behavioural pattern in four distinct sharks when present in open, oceanic waters. Our observations suggest that adult migrating tope, at least to a certain extent, display clear diurnal vertical migrations into mesopelagic layers and follow the DVM of potential prey species when in oceanic areas. Quantification of how common this behaviour is in tope requires a larger sampling size than is currently available. Previously, both conventional and electronic-tagging studies as well as our own results have shown that not all adult tope undertake far ranging migrations into oceanic areas^28–31^. In fact, the results from geolocation estimates presented here corroborate the enormous plasticity in habitat use and migration behaviour of (adult) tope, considering, that only a quarter of all tagged specimens displayed the migrations and behaviour described above. The remaining sharks largely stayed on the continental shelf or on the shelf slopes. Accordingly, more extensive electronic tagging programs, ideally with concurrent acoustic surveys clearly mapping out the availability of prey fields within the tope roaming region, are needed to further define the regularity of the (meso)pelagic habitat use by tope and their characterization as a “semi-oceanic” species based on the frequent, albeit rather unquantified occurrence of adult sharks in open ocean areas.

Throughout their distribution range, tope have been subject to targeted and incidental fisheries with a variety of gears for a long time. In the Northeast Atlantic, tope are mainly landed from French mixed fisheries in the English Channel and Celtic Seas as well as from bottom longline fisheries on the Azores^66^. Tope are particularly vulnerable to overfishing, and significant population declines in the southern North Sea have been observed^67^. Globally, tope have only recently been re-assessed from “Vulnerable” to “Critically Endangered”^25^ and have been included in Appendix II of the Convention on the Conservation of Migratory Species of Wild Animals. While the EU effectively prohibits longline fisheries for tope and landings of this species from longline fisheries in large parts of Union waters and international waters, no such regulations apply for other gears there. This study's findings indicate that the habitat expansion of adult tope into mesopelagic layers of the high seas further increases their risk of incidental fisheries capture. This is already evident in comparatively high bycatch of tope from midwater trawl gears employed in fisheries targeting herring, horse mackerel or blue whiting^68^—large scale fisheries often operating in deep layers of the open ocean without regulations on tope bycatch.

## Methods

### Data collection

#### Tagging data

Tagging work conducted for this study was reviewed and approved by the Ministry of Energy, Agriculture, the Environment, Nature and Digitalization of the Government of Schleswig–Holstein, Germany, department Animal Welfare, permission V241-23762/2017(56-4/17) and the Thünen Institute, Bremerhaven, Germany or by Marine Scotland Science. The tagging procedures were performed in accordance with guidelines and regulations on animal experiments as stated in the corresponding permits, and all field work was conducted in compliance with the ARRIVE guidelines. Tope were caught by angling from recreational fishing boats using individual baited rod and line. Hooks were baited with Atlantic mackerel (*Scomber scombrus*) and deployed near the seafloor. Angling and tagging took place in Luce Bay, southwest Scotland (54.7° N, 4.7° W) in October 2015 (n = 1) as well as near Helgoland Island in the German Bight of the North Sea (54.3° N, 7.9° E) in August and September 2018–2020 (n = 15). Caught specimens were brought on board for tagging. Total length (TL) and sex were recorded. All specimens were fitted with MiniPAT pop-off satellite archival tags (PSAT, Wildlife Computers Inc). In the Scottish specimen, the tag was fixed intramuscularly with a titanium plate inserted into the dorsal musculature next to the first dorsal fin using a sterilized stainless-steel applicator and attached via a 5 cm monofilament leader (200 lb) (for more details on capture, handling and tagging procedures, see Ref.^30^). The specimens from the German Bight were restrained through placing a dark towel over the head. The PSATs were attached through a 4.5 mm hole punched into the first dorsal fin near the anterior base with sterilized punch pliers. The tags were fixed with a 30 cm monofilament leader (250 lb) fed through a 4.5 mm medicinal silicone tube (20 cm) to prevent fraying of the fin. The tether was fed through the hole and crimped behind the dorsal fin with a stainless double sleeve crimp, creating a loop with the PSAT trailing median behind the first dorsal fin. All tags were marked with contact details. Handling times of the individual sharks were kept as short as possible and ranged from 5 to 15 min (total time from taking the bait until released back into the water). All sharks were released at their capture site.

The tags were programmed to record time series of depth, temperature and ambient light. The tag deployed in Scotland was pre-programmed to release after 180 days of deployment and a sample interval of 5 min (with summarized messages created for every 24 h). The tags deployed in the German Bight were pre-programmed to release after 270 days of deployment at a sample interval of 6 min for all parameters. To increase deployment duration, an on–off–on sampling schedule was programmed with 2 days on, 1 day off after 70 days of initial deployment and continuous sampling at the preset sample interval. No daily messages were generated.

After detaching from the sharks, the tags surfaced and transmitted the time series and message data via the ARGOS satellite network link. Based on the data transmissions received after detachment of the tags, four of the sharks showed exceptional behaviour regarding migration range and depth use that stood out from the other transmissions. In the present study, these four tags transmitting conspicuous data are considered (1 from Scotland, 3 from Germany).

#### Hydroacoustic and auxiliary data

Hydroacoustic data used for identifying deep scattering layers (DSL) and DVM patterns were recorded in March/April 2016 on an east–west transect at circa 58° N in the Rockall Trough during the ICES-coordinated “International Blue Whiting Spawning Survey” (IBWSS) west of the British Isles on board the Dutch FRV “Tridens” and in June 2018 during the “Biomass and Active Flux in the Bathypelagic Zone” (BATHYPELAGIC) survey on board the Spanish RV “Sarmiento de Gamboa” in the Northeast Atlantic on 20° W in the area of the permanent Azores Front at circa 38° N (for more information, see Ref.^48^) (Fig. 1). Both vessels used calibrated, hull mounted EK60 scientific echosounders (Kongsberg Simrad AS, Kongsberg), operated in continuous wave mode at centre frequencies of 18 (12° half-way beam opening) and 38 (7° half-way beam opening) kHz. All transducers were operated at 2000 W. Pulse duration was fixed at 1.024 ms, with a variable ping interval fixed at 2.5 or 5 s respectively, where conditions allowed. In both areas, hydroacoustic data were collected while the vessel was moving at a normal survey speed of approximately 10 knots. A representative section of a 24-h cycle was selected from each dataset to elucidate diurnal vertical migration patterns of mesopelagic organisms in the corresponding areas.

### Data analysis

#### Migration paths and habitat use

Geolocation of time series data from the four tags was conducted using the Wildlife Computers GPE3 state-space model taking advantage of observations of twilight, sea surface temperature (SST) and dive depth from the tag data. SST values were validated through comparison with observational data of SST (NOAA OI SST V2 High Resolution dataset^69^) and a bathymetry reference dataset (ETOPO1-Bedrock^70^) was used to add seafloor depth information. The model incorporates a movement model based on user defined swimming speed parameters^71^. Maximum likelihood positions are estimated through a gridded hidden Markov Model with a 0.25 by 0.25° grid spacing, and associated location probabilities of 95% were estimated. Multiple swimming speeds were considered. Model runs with 1.5 ms^−1^ swimming speed yielded the highest location scores and were deemed appropriate. Different phases (coastal/continental vs. oceanic) were allocated where individuals crossed the continental shelf break, i.e. swam over the continental slope into oceanic waters (location processing) and depth measurements from the time series exceeded 250 m for the first time (to account for given uncertainties in the geolocation methods possibly not providing the exact date of this transition). Phases towards the end of the deployment with residency periods (of one shark) in spatially restricted areas of highly variable bottom depths (including both coastal and oceanic regions) were excluded from this classification to account for location uncertainties. Further analyses of time series data (profiles of Time at Depth, TAD) from the tags were conducted using the Cran-R 4.0.0 environment and the RchivalTag library^72^. Periodicity of the vertical migration patterns of tope was assessed through wavelet and autocorrelation analysis^73,74^. Wavelet analysis requires a regularly spaced dataset. For this purpose, the depth location versus time dataset was resampled to an hourly resolution. Missing points were interpolated through GAM splines based on a representative subset of the data surrounding, missing values.

The general observed depth use pattern of the four sharks during the oceanic phase was simplified through a Generalized Additive Mixed Model (GAMM)^44,75,76^ with an autocorrelation structure of order 1: ${Z}_{shark }\sim s\left(Time, k = 5, bs = 'cc'\right)+Ptt$, where Z_shark_ is the depth location of the shark at any given time expressed in rounded hours (Time), k defines the degrees of freedom and s is the Gaussian smoother function and *Ptt* is the tag or shark identifier, entering the model as a random effect variable. A summarizing model comprising all dives of all 4 sharks was evaluated. Significant differences between the individual sharks were detected (p < 0.01), hence for each shark, an individual model was developed.

#### Hydroacoustic data

Acoustic backscatter information was translated into Scattering volume data (Sv, dB re m^−1^), a commonly used logarithmic measure of acoustic density, the received acoustic energy scaled by the ensonified volume. The Sv data was processed in 6 min × 1 m bins, which can be visualized as composite echograms (Fig. 4). A synthetic variable named Δ_Sv_ was created by subtracting Sv at 38 kHz from Sv at 18 kHz (equivalent to a fraction in linear space), providing a helpful metric to identify structural differences in the depth strata^52^.

The resulting synthetic variable was utilized to characterize and classify functional groups of mesopelagic scatterers following a classification tree based on the size and scattering properties of different organism groups found in mesopelagic layers^53,55^. Sv was categorised into four groups with corresponding properties: Small swimbladdered fishes (including small non-swimbladdered fishes and crustaceans; Sm.(N)SB/Crust.) where − 14 dB < Δ_Sv18kHz−38 kHz_ <  − 3 dB; large non-swimbladdered fishes (Lrg.NSB) where − 3 dB < Δ_Sv18 kHz−38 kHz_ < 0 dB; gelatinous zooplankton, cephalopods and pteropods (FL) where 0 dB < Δ_Sv18 kHz−38 kHz_ < 3 dB and large swimbladdered fishes (Lrg.SB) where 3 dB < Δ_Sv18 kHz−38 kHz_ < 12 dB.

To get a better impression of the diel distribution of the four acoustic classes, the class specific 2D kernel density distributions were visualized (Figs. 5, 6). The 2D kernel density can be summarized as a nonparametric probability density function, here displaying the probability of occurrence of a class at a given depth and time of the day, scaled to 1.

## Supplementary Information

Supplementary Figures.

## References

[1] Angel MV (1993). Biodiversity of the Pelagic Ocean. Conserv. Biol..

[2] Irigoien X (2014). Large mesopelagic fishes biomass and trophic efficiency in the open ocean. Nat. Commun..

[3] Hays GC (2003). A review of the adaptive significance and ecosystem consequences of zooplankton diel vertical migrations. Hydrobiology.

[4] Klevjer TA (2016). Large scale patterns in vertical distribution and behaviour of mesopelagic scattering layers. Sci. Rep..

[5] Hammerschlag N, Gallagher AJ, Lazarre DM (2011). A review of shark satellite tagging studies. J. Exp. Mar. Biol. Ecol..

[6] Dulvy NK (2008). You can swim but you can't hide: The global status and conservation of oceanic pelagic sharks and rays. Aquat. Conserv..

[7] Pacoureau N (2021). Half a century of global decline in oceanic sharks and rays. Nature.

[8] Compagno, L. J. V. Pelagic elasmobranch diversity. In *Sharks of the Open Ocean*, 14–23 (2008).

[9] Howey LA (2016). Into the deep: The functionality of mesopelagic excursions by an oceanic apex predator. Ecol. Evol..

[10] Francis MP (2018). Oceanic nomad or coastal resident? Behavioural switching in the shortfin mako shark (*Isurus oxyrinchus*). Mar. Biol..

[11] Skomal G (2021). Horizontal and vertical movement patterns and habitat use of juvenile porbeagles (*Lamna nasus*) in the western north Atlantic. Front. Mar. Sci..

[12] Gaube P (2018). Mesoscale eddies influence the movements of mature female white sharks in the Gulf Stream and Sargasso Sea. Sci. Rep..

[13] Coelho R, Fernandez-Carvalho J, Santos MN (2015). Habitat use and diel vertical migration of bigeye thresher shark: Overlap with pelagic longline fishing gear. Mar. Environ. Res..

[14] Arostegui MC (2020). Vertical movements of a pelagic thresher shark (*Alopias pelagicus*): Insights into the species' physiological limitations and trophic ecology in the Red Sea. Endanger. Species Res..

[15] Coffey DM, Carlisle AB, Hazen EL, Block BA (2017). Oceanographic drivers of the vertical distribution of a highly migratory, endothermic shark. Sci. Rep..

[16] Coffey DM, Royer MA, Meyer CG, Holland KN (2020). Diel patterns in swimming behavior of a vertically migrating deepwater shark, the bluntnose sixgill (*Hexanchus griseus*). PLoS One.

[17] Francis MP, Holdsworth JC, Block BA (2015). Life in the open ocean: Seasonal migration and diel diving behaviour of Southern Hemisphere porbeagle sharks (*Lamna nasus*). Mar. Biol..

[18] Jorgensen SJ (2012). Eating or meeting? Cluster analysis reveals intricacies of white shark (*Carcharodon carcharias*) migration and offshore behavior. PLoS One.

[19] Nelson DR (1997). An acoustic tracking of a megamouth shark, *Megachasma pelagios*: A crepuscular vertical migrator. Environ. Biol. Fish..

[20] Sims DW, Southall EJ, Tarling GA, Metcalfe JD (2005). Habitat-specific normal and reverse diel vertical migration in the plankton-feeding basking shark. J. Anim. Ecol..

[21] Watanabe YY, Papastamatiou YP (2019). Distribution, body size and biology of the megamouth shark *Megachasma pelagios*. J. Fish Biol..

[22] Braun CD, Skomal GB, Thorrold SR (2018). Integrating archival tag data and a high-resolution oceanographic model to estimate basking shark (*Cetorhinus maximus*) movements in the Western Atlantic. Front. Mar. Sci..

[23] Jorgensen SJ (2010). Philopatry and migration of Pacific white sharks. Proc. R. Soc. B.

[24] Lipscombe RS (2020). Habitat use and movement patterns of tiger sharks (*Galeocerdo cuvier*) in eastern Australian waters. ICES J. Mar. Sci..

[25] Walker, T. I. *et al. Galeorhinus galeus*. The IUCN Red List of Threatened Species 2020: e.T39352A2907336. 10.2305/IUCN.UK.2020-2.RLTS.T39352A2907336.en (2020). (Downloaded on 18 June 2021).

[26] Chabot CL (2015). Microsatellite loci confirm a lack of population connectivity among globally distributed populations of the tope shark *Galeorhinus galeus* (Triakidae). J. Fish Biol..

[27] Bester-van der Merwe AE (2017). Population genetics of Southern Hemisphere tope shark (*Galeorhinus galeus*): Intercontinental divergence and constrained gene flow at different geographical scales. PLoS One.

[28] Stevens JD (1990). Further results from a tagging study of pelagic sharks in the north-east Atlantic. J. Mar. Biol. Assoc. UK.

[29] West GJ, Stevens JD (2001). Archival tagging of school shark, *Galeorhinus galeus*, in Australia: Initial results. Environ. Biol. Fish..

[30] Thorburn J (2019). Ontogenetic variation in movements and depth use, and evidence of partial migration in a Benthopelagic Elasmobranch. Front. Ecol. Evol..

[31] McMillan MN, Huveneers C, Semmens JM, Gillanders BM (2019). Partial female migration and cool-water migration pathways in an overfished shark. ICES J. Mar. Sci..

[32] Walker T (1999). *Galeorhinus galeus* fisheries of the World, in: Case studies of management of elasmobranch fisheries. FAO Fish. Tech. Pap..

[33] Brown L, Bridge N, Walker T (2000). Summary of tag releases and recaptures in the Southern Shark Fishery. Mar. Freshw. Resour. Inst. Rep..

[34] Lucifora L, Menni R, Escalante A (2004). Reproductive biology of the school shark, *Galeorhinus galeus*, off Argentina: Support for a single south western Atlantic population with synchronized migratory movements. Environ. Biol. Fish..

[35] Jaureguizar AJ, Argemi F, Trobbiani G, Palma ED, Irigoyen AJ (2018). Large-scale migration of a school shark, *Galeorhinus galeus,* in the Southwestern Atlantic. Neotrop. Ichthyol..

[36] Nosal AP (2021). Triennial migration and philopatry in the critically endangered soupfin shark *Galeorhinus galeus*. J. Appl. Ecol..

[37] Cuevas J, Garcia M, Di Giacomo E (2014). Diving behaviour of the critically endangered tope shark *Galeorhinus galeus* in the Natural Reserve of Bahia San Blas, northern Patagonia. Anim. Biotelemetry.

[38] Iosilevskii G, Papastamatiou YP, Meyer CG, Holland KN (2012). Energetics of the yo-yo dives of predatory sharks. J. Theor. Biol..

[39] Carey FG, Scharold JV, Kalmijn AJ (1990). Movements of blue sharks (*Prionace glauca*) in depth and course. Mar. Biol..

[40] Nakamura I, Watanabe YY, Papastamatiou YP, Sato K, Meyer CG (2011). Yo-yo vertical movements suggest a foraging strategy for tiger sharks *Galeocerdo cuvier*. Mar. Ecol. Prog. Ser..

[41] Thorrold SR (2014). Extreme diving behaviour in devil rays links surface waters and the deep ocean. Nat. Commun..

[42] Braun CD, Gaube P, Sinclair-Taylor TH, Skomal GB, Thorrold SR (2019). Mesoscale eddies release pelagic sharks from thermal constraints to foraging in the ocean twilight zone. Proc. Nat. Acad. Sci..

[43] Andrzejaczek S, Gleiss AC, Pattiaratchi CB, Meekan MG (2019). Patterns and drivers of vertical movements of the large fishes of the epipelagic. Rev. Fish. Biol. Fish..

[44] Papastamatiou YP (2015). Drivers of daily routines in an ectothermic marine predator: Hunt warm, rest warmer?. PLoS One.

[45] Proud R, Cox MJ, Brierley AS (2017). Biogeography of the global ocean’s mesopelagic zone. Curr. Biol..

[46] Sutton TT (2017). A global biogeographic classification of the mesopelagic zone. Deep Sea Res..

[47] Ariza A (2016). Vertical distribution, composition and migratory patterns of acoustic scattering layers in the Canary Islands. J. Mar. Syst..

[48] Peña M, Cabrera-Gámez J, Domínguez-Brito AC (2020). Multi-frequency and light-avoiding characteristics of deep acoustic layers in the North Atlantic. Mar. Environ. Res..

[49] Peña M (2014). Acoustic detection of mesopelagic fishes in scattering layers of the Balearic Sea (western Mediterranean). Can. J. Fish. Aquat. Sci..

[50] Menkes CE (2015). Seasonal oceanography from physics to micronekton in the south-west Pacific. Deep Sea Res..

[51] Urmy SS, Horne JK (2016). Multi-scale responses of scattering layers to environmental variability in Monterey Bay, California. Deep Sea Res..

[52] Korneliussen RJ (2018). Acoustic target classification. ICES Coop. Res. Rep..

[53] D'Elia M (2016). Diel variation in the vertical distribution of deep-water scattering layers in the Gulf of Mexico. Deep Sea Res..

[54] Scoulding B, Chu D, Ona E, Fernandes PG (2015). Target strengths of two abundant mesopelagic fish species. J. Acoust. Soc. Am..

[55] Geoffroy M (2019). Mesopelagic sound scattering layers of the high arctic: Seasonal variations in biomass, species assemblage, and trophic relationships. Front. Mar. Sci..

[56] Shea EK, Vecchione M (2010). Ontogenic changes in diel vertical migration patterns compared with known allometric changes in three mesopelagic squid species suggest an expanded definition of a paralarva. ICES J. Mar. Sci..

[57] Lucifora LO, Garcia VB, Menni RC, Escalante AH (2006). Food habits, selectivity, and foraging modes of the school shark *Galeorhinus galeus*. Mar. Ecol. Prog. Ser..

[58] Morato T, Sola E, Gros MP, Menezes G (2003). Diets of thornback ray (*Raja clavata*) and tope shark (*Galeorhinus galeus*) in the bottom longline fishery of the Azores, northeastern Atlantic. Fish. Bull..

[59] Ellis JR, Pawson MG, Shackley SE (1996). The comparative feeding ecology of six species of shark and four species of ray (Elasmobranchii) in the North-East Atlantic. J. Mar. Biol. Assoc. UK..

[60] Clarke MR, Clarke DC, Martins HR, Silva HM (1996). The diet of blue shark (*Prionace glauca*) in Azorean waters, *Arquipélago*. Life Mar. Sci..

[61] Bond ME, Tolentino E, Mangubhai S, Howey LA (2015). Vertical and horizontal movements of a silvertip shark (*Carcharhinus albimarginatus*) in the Fijian archipelago. Anim. Biotelemetry.

[62] Saba GK (2021). Toward a better understanding of fish-based contribution to ocean carbon flux. Limnol. Oceanogr..

[63] Arkhipkin AI (2013). Squid as nutrient vectors linking Southwest Atlantic marine ecosystems. Deep Sea Res..

[64] Bird CS (2018). A global perspective on the trophic geography of sharks. Nat. Ecol. Evol..

[65] Spaet JLY, Lam CH, Braun CD, Berumen ML (2017). Extensive use of mesopelagic waters by a Scalloped hammerhead shark (*Sphyrna lewini*) in the Red Sea. Anim. Biotelemetry.

[66] ICES (2020). Working Group on Elasmobranch Fishes (WGEF). ICES Sci. Rep..

[67] Murgier J (2021). Rebound in functional distinctiveness following warming and reduced fishing in the North Sea. Proc. R. Soc. B.

[68] Pastoors, M. A., van Helmond, E. B., van Marlen, B., van Overzee, H. & de Graaf, E. Pelagic pilot project discard ban, 2013–2014. (IMARES, Wageningen UR, Report Number C071/14) (2014).

[69] Reynolds RW (2007). Daily high-resolution-blended analyses for sea surface temperature. J. Clim..

[70] NOAA National Geophysical Data Center. *ETOPO1 1 Arc-Minute Global Relief Model*. (NOAA National Centers for Environmental Information, 2009).

[71] Pedersen MW, Patterson TA, Thygesen UH, Madsen H (2011). Estimating animal behaviour and residency from movement data. Oikos.

[72] Bauer, R. RchivalTag: Analyzing Archival Tagging Data. A set of functions to generate, access and analyze standard data products from archival tagging data. (2020). https://cran.r-project.org/package=RchivalTag. Accessed on 8 November 2021.

[73] Cazelles B (2008). Wavelet analysis of ecological time series. Oecologia.

[74] Venables, W. N. & Ripley, B. D. *Modern Applied Statistics with S*, 4th ed. (Springer, 2002). 10.1007/978-0-387-21706-2.

[75] Wood, S. mgcv: Mixed GAM Computation Vehicle with GCV/AIC/REML smoothness estimation and GAMMs by REML/PQL (2012). https://cran.r-project.org/package=mgcv. Accessed on 8 November 2021.

[76] Wood S. N. *Generalized Additive Models. An Introduction with R.* 2nd ed. (Chapman & Halll, 2017). 10.1201/9781315370279.

