# Supplementary material for: Extensive oceanic mesopelagic habitat use of a migratory continental shark species
Source: Sci Rep. 2022 Feb 7;12:2047. doi: 10.1038/s41598-022-05989-z (PMC8821621; doi:10.1038/s41598-022-05989-z)
Supplement: Supplementary file 1 — Supplementary Figures. [file 41598_2022_5989_MOESM1_ESM.docx]

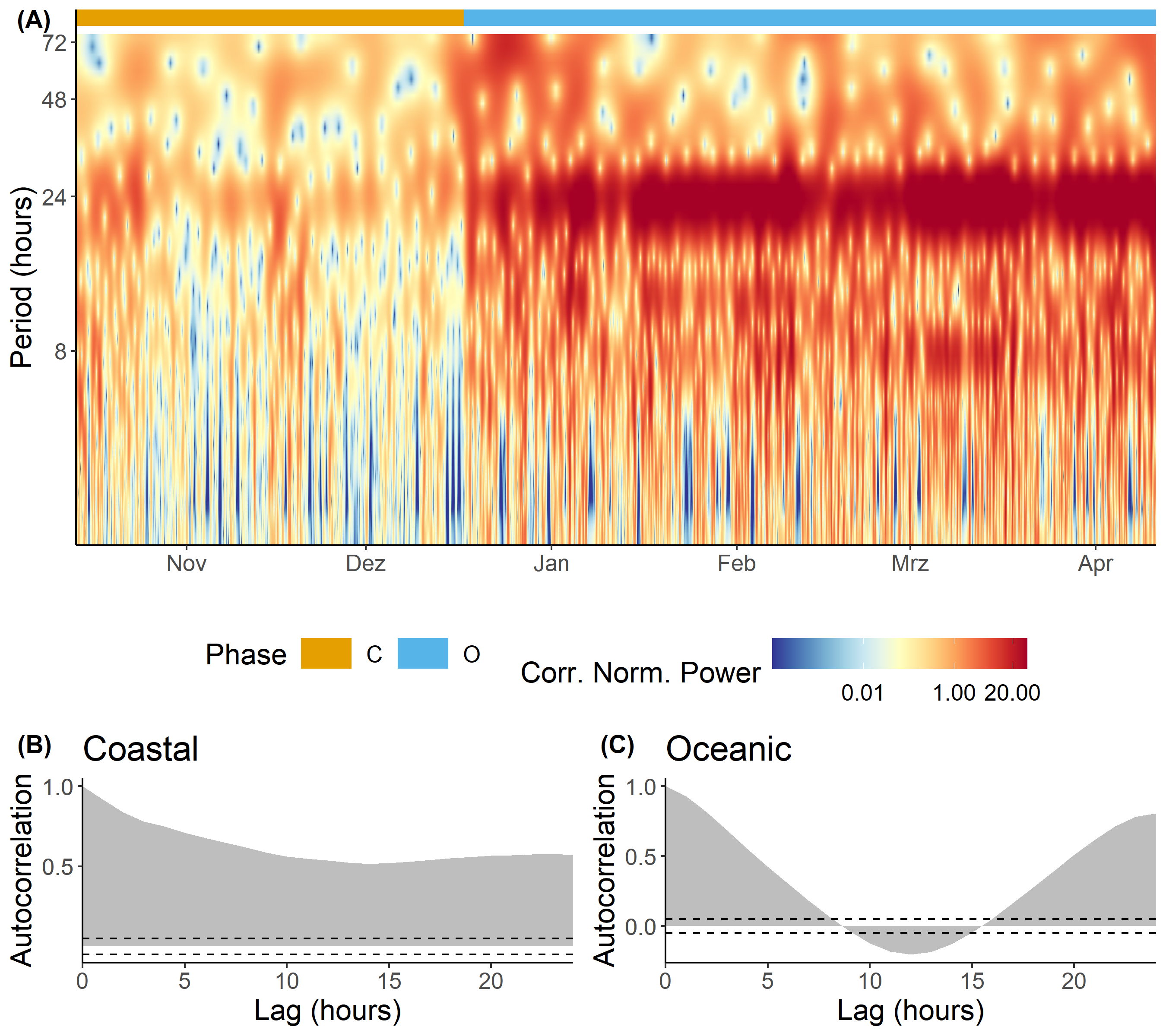


Suppl. figure 1 Bias-corrected power normalized by the variance spectrum of the wavelet function (A) for shark 153233 and the autocorrelation function for the period spent in Coastal (B) or Oceanic (C) waters


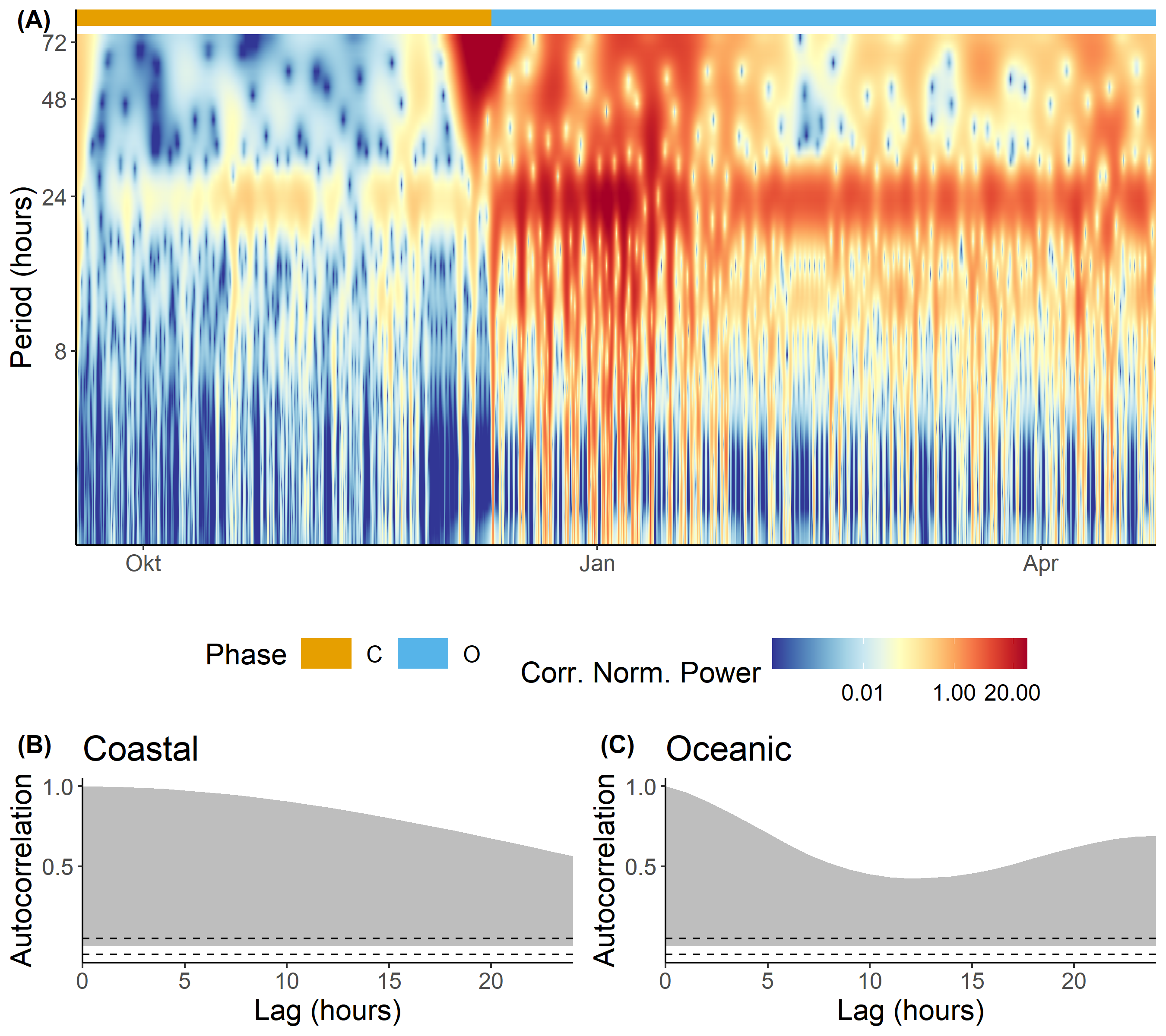


Suppl. figure 2 Bias-corrected power normalized by the variance spectrum of the wavelet function (A) for shark 168495 and the autocorrelation function for the period spent in Coastal (B) or Oceanic (C) waters


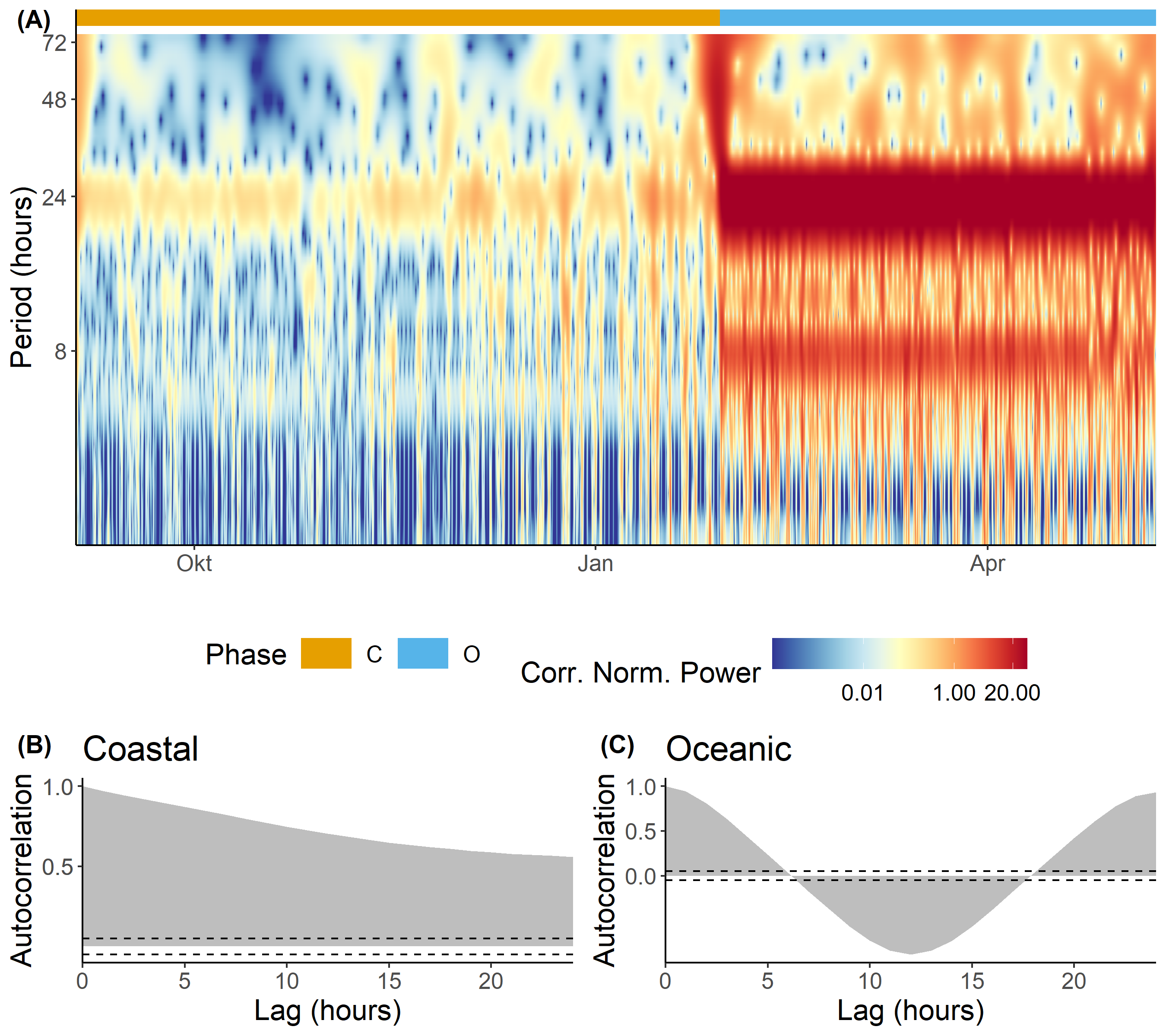


Suppl. figure 3 Bias-corrected power normalized by the variance spectrum of the wavelet function (A) for shark 168499 and the autocorrelation function for the period spent in Coastal (B) or Oceanic (C) waters


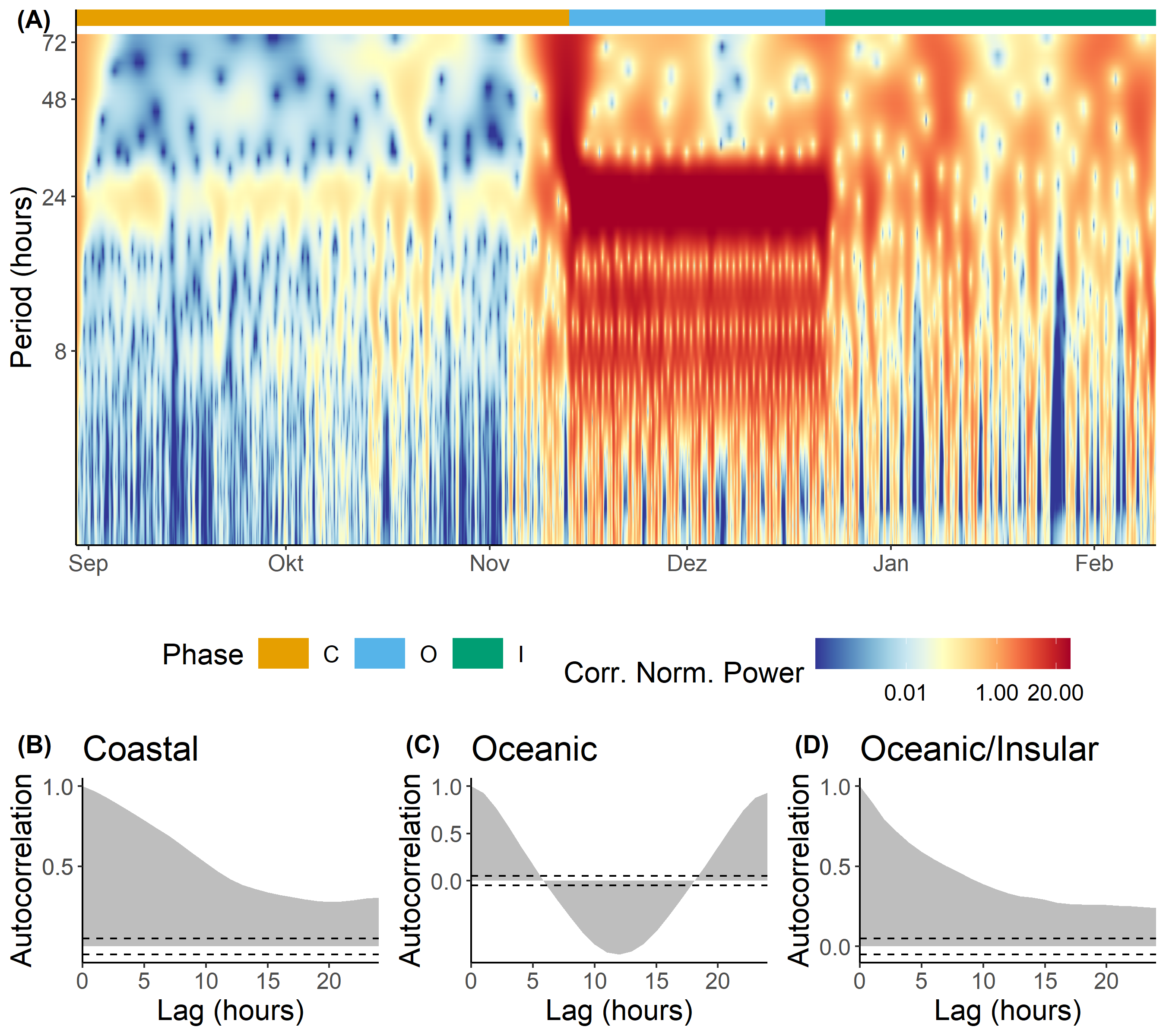


Suppl. figure 4 Bias-corrected power normalized by the variance spectrum of the wavelet function (A) for shark 168500 and the autocorrelation function for the period spent in Coastal (B), Oceanic (C) and (towards end of deployment) Oceanic/Insular (D) waters
